# A Staging Auxiliary Diagnosis Model for Nonsmall Cell Lung Cancer Based on the Intelligent Medical System

**DOI:** 10.1155/2021/6654946

**Published:** 2021-02-08

**Authors:** Jia Wu, Fangfang Gou, Yanlin Tan

**Affiliations:** ^1^School of Computer Science and Engineering, Central South University, Changsha 410083, China; ^2^PET-CT Center, The Second Xiangya Hospital of Central South University, Changsha 410083, China

## Abstract

At present, human health is threatened by many diseases, and lung cancer is one of the most dangerous tumors that threaten human life. In most developing countries, due to the large population and lack of medical resources, it is difficult for doctors to meet patients' needs for medical treatment only by relying on the manual diagnosis. Based on massive medical information, the intelligent decision-making system has played a great role in assisting doctors in analyzing patients' conditions, improving the accuracy of clinical diagnosis, and reducing the workload of medical staff. This article is based on the data of 8,920 nonsmall cell lung cancer patients collected by different medical systems in three hospitals in China. Based on the intelligent medical system, on the basis of the intelligent medical system, this paper constructs a nonsmall cell lung cancer staging auxiliary diagnosis model based on convolutional neural network (CNNSAD). CNNSAD converts patient medical records into word sequences, uses convolutional neural networks to extract semantic features from patient medical records, and combines dynamic sampling and transfer learning technology to construct a balanced data set. The experimental results show that the model is superior to other methods in terms of accuracy, recall, and precision. When the number of samples reaches 3000, the accuracy of the system will reach over 80%, which can effectively realize the auxiliary diagnosis of nonsmall cell lung cancer and combine dynamic sampling and migration learning techniques to train nonsmall cell lung cancer staging auxiliary diagnosis models, which can effectively achieve the auxiliary diagnosis of nonsmall cell lung cancer. The simulation results show that the model is better than the other methods in the experiment in terms of accuracy, recall, and precision.

## 1. Introduction

Lung cancer is the malignant tumor with the highest incidence (11.6%) and mortality (18.4%) in the world [1]. In 2018, there were approximately 2.0939 million new lung cancers worldwide, and 1.761 million died of lung cancer [2]. More than half of lung cancers worldwide occur in developing or underdeveloped countries [3]. China is one of the developing countries, and its number of lung cancer incidences and deaths has ranked first among malignant tumors for 10 consecutive years [4]. Approximately 787,000 new lung cancer patients are added each year, and the number of deaths due to lung cancer has reached 631,000, accounting for about a quarter of China's deaths from malignant tumors [5]. Lung cancer has become one of the high-risk tumors that cause human deaths. Improving the survival rate of lung cancer has become a major problem that needs to be solved urgently worldwide [1].

Lung cancer is divided into nonsmall cell lung cancer (NSCLC) and small cell lung cancer according to the pathological characteristics that form its tissue; more than 80% of lung cancer patients belong to NSCLC [6]. Because cancer cells are not easy to find in the human body, most lung cancer patients have entered the middle and advanced stages when they are diagnosed [7]. At this time, the possibility of cure is very low, and the overall 5-year survival rate is only 15% [8]. However, if it can be detected and treated early, the 5-year survival rate can be increased to 70% [8]. “Early detection, early diagnosis and early treatment” are effective means to improve the survival rate of lung cancer [7].

However, in many developing countries, limited medical resources cannot meet the needs of patients for medical treatment, taking China as an example. On the one hand, the total amount of medical resources is insufficient. According to statistics, China's population accounts for 22% of the world's population, but medical resources account for only 2% of the world's medical resources [9]. On average, more than 1,000 patients can only share one doctor. On the other hand, medical resources are unevenly distributed. Most high-quality resources are concentrated in large cities and developed regions. The urban population, which accounts for only 30% of the total population, enjoys 80% of the medical and health resources, while 70% of the rural population only enjoys 20% of the medical resources [10].

Developing countries are facing difficult dilemmas in the prevention, examination, and treatment of lung cancer.

1. With limited medical technology, lung cancer patients' condition is difficult to detect at an early stage. Most patients are already in the middle and advanced stages when they are diagnosed, so the possibility of cure is very small
2. The number of illnesses is large and the number of doctors is small. The imbalance of the doctor-patient ratio leads to a large workload and long working hours for doctors, which increases the possibility of misdiagnosis and missed diagnosis
3. The doctor-patient relationship is strained. Patients lack professional medical knowledge, and doctors cannot take care of all patients, leading to a trust crisis between the two
4. Medical resources are scarce and treatment costs are high. Many patients cannot afford it, and they have to delay treatment or even give up treatment

In order to solve the problems of limited technology and insufficient resources faced by medical systems in developing countries, artificial intelligence systems are gradually used as auxiliary tools to improve the current medical conditions in developing countries [11]. On the basis of massive medical data, the intelligent assistance system allows machines to learn expert knowledge, thereby providing professional and intelligent diagnosis solutions to doctors [12]. Doctors can use it to quickly assess the health of patients. In addition, the feedback results from doctors during use can further optimize the system [11].

The symptoms, treatment, and prognosis of each clinical stage of NSCLC patients are different, so it is very necessary to determine the stage of the patient to choose a treatment plan [13]. An effective assistance system can extract appropriate patient characteristics as the basis for research to achieve a more accurate diagnosis. Especially in the diagnosis and decision-making of lung cancer, it can reduce the possibility of doctors' misdiagnosis and missed diagnosis.

However, in many current works, when the patient's medical records are converted into patient features, a lot of important information is lost. At the same time, due to the different lengths of semantic information in the medical records, the extraction of patient data features is not accurate enough. The most important is, in the NSCLC data set, due to the imbalance of the proportion of positive and negative cases, the diagnosis of NSCLC has a bias problem.

Based on the above problems, this paper constructs a nonsmall cell lung cancer staging auxiliary diagnosis model (CNNSAD) on the basis of computer-aided diagnosis and an intelligent medical system. This method converts the patient's medical record into a sequence of words and uses a convolutional neural network (CNN) to automatically extract the text information in the patient's medical record, so as to train the NSCLC staging auxiliary diagnosis model to realize the auxiliary diagnosis of NSCLC staging. At the same time, for knowledge transfer problems and small sample disease training problems, the CNNSAD model introduces dynamic sampling technology to construct a balanced data set and uses the model's diagnostic results on different samples to dynamically think about the sample sampling probability. This ensures that CNNSAD can pay more attention to misclassified samples and samples with low classification confidence, thereby improving the effect of model diagnosis.

The main contributions of this article are as follows:

1. The NSCLC staging auxiliary diagnosis model proposed in this article is based on the diagnosis of whether the patient has lung cancer, it can diagnose the clinical stage of the patient. Among them, the convolutional neural networks can extract text semantic information of different lengths through different convolution kernels to improve the diagnostic performance of the model
2. The CNNSAD model incorporates dynamic sampling and transfer learning technology. The dynamic sampling technique separates sampling on the positive and negative samples to improve the impact of the imbalance in the data of patients with different stages of NSCLC and to a certain extent avoid the inability to obtain sufficient information when the sample is insufficient. The transfer learning technology realizes the knowledge sharing and transfer between different disease models and to a certain extent avoids the shortcomings of not being able to obtain sufficient information in the case of insufficient samples
3. This article is based on the information of 8920 NSCLC patients from three hospitals in China for experimental. The results show that the system has better performance in the auxiliary diagnosis of NSCLC staging. Doctors use the results of the auxiliary system as a reference for the second diagnosis, which can improve the accuracy and efficiency of the diagnosis

## 2. Related Work

Artificial intelligence medical decision-making system has become a research hotspot in the medical field. Many research methods are widely used in the medical field.

Logistic regression is not easy to overfit and is often used in predictive models of decision-making systems. However, it is difficult to deal with nonlinear problems and the interaction between variables and is only suitable for simple linear problems. Zhang et al. [14] constructed a logistic regression decision model to predict the hospitalization or transfer of patients after the first triage to the emergency department (ED). Graham et al. [15] also developed a logistic model regression in the decision-making system to predict the hospitalization rate, helping the hospital to make advance resource planning and avoid patient flow bottlenecks.

Support vector machine (SVM) has good generalization ability and is robust to high-dimensional data. Therefore, it is widely used in the medical field. However, it is very sensitive to uncertainty and is prone to overfitting in high-dimensional spaces. Khachane [16] used support vector machines to classify brain magnetic resonance imaging MRI and knee MRI medical images and achieved good results. Chen et al. [17] also proposed a support vector machine- (SVM-) based risk prediction system to predict the risk of preterm birth. Provide care for women who may have pregnancy-related problems, thereby improving the health of pregnant women and fetuses. Similarly, Baek et al. [18] developed an SVM-based liver classifier to distinguish liver conditions, including normal, low-fat fibrosis, high-fat fibrosis, and inflammation. To determine the comparison between normal liver and diseased liver, it provides a new starting point for a coherent framework in scattering features or clustering in multiparameter space.

Naive Bayes classifier (NB) also has its own competitiveness in the medical field. Wang et al. [19] used Bayesian classifiers in the diagnosis of chronic diseases. The model can stably calculate the probability of chronic diseases, which is of great significance for predicting chronic diseases. Altayeva et al. [20] developed a heart disease decision diagnosis system (HDPS) based on fusion Bayesian and *K*-means clustering algorithm, which can effectively improve the accuracy of patient diagnosis.

A fuzzy inference system (FIS) makes up for the shortcomings of Naive Bayes that is very sensitive to the expression of input data and has gradually become a common method in medical decision-making systems. Cui et al. [21] proposed the application of a fuzzy inference system to diagnose tuberculosis, which can diagnose patients with high or low risk of tuberculosis. The fuzzy rule computer decision system proposed by Mendez et al. [22] uses the heuristic knowledge provided by clinicians on the basis of diagnostic classification to effectively realize the automatic adjustment of drug dosage according to the needs of patients. Liu et al. [23] proposed a new data decision model for developing prostate cancer in developing countries using fuzzy reasoning logic and constructed an intelligent medical system integrating disease detection, medical data analysis and fusion, treatment recommendation, and risk management.

Neural networks are robust to noisy data, can represent complex functions, and become the most commonly used tool in decision-making systems. Kwasigroch et al. [24] proposed a decision support system based on convolutional neural networks to automatically screen diabetic retinopathy. The system can not only diagnose diabetic retinopathy but also its current stage. Harjai and Khatri [25] provide early diagnosis of cardiovascular disease through an artificial neural network-based intelligent clinical decision support system (CDSS). This model helps doctors diagnose the patient's condition and choose a suitable treatment for the patient. Thereby reducing the cost and effort required to prescribe unnecessary treatments. Similarly, Wu et al. [26] proposed a neural network-based intelligent decision support system for the early diagnosis of malignant melanoma. The system uses ten neural networks to work in parallel. Compared with a single neural network, this system structure effectively improves work efficiency.

In summary, the classification accuracy of deep neural networks is high; it has become a widely used model in machine learning and has achieved advanced results in many tasks. Based on this, this research is based on the convolutional neural network, fusing dynamic sampling and migration learning technology to realize the auxiliary diagnosis of nonsmall cell lung cancer staging.

## 3. System Model

### 3.1. The Overall Framework of the CNNSAD Model

In smart cities, the smart medical system, as an auxiliary tool in the medical field, has enabled medicine to enter a new visual information era, thereby alleviating the medical difficulties and expensive problems of the masses [27]. With the development of the Internet of Things and 5G technology, machine-assisted systems have played an increasingly important role in the mining, classification, and decision-making of medical information [28]. Therefore, we have constructed an auxiliary diagnosis model based on a convolutional neural network for NSCLC staging on the basis of computer-aided diagnosis and intelligent medical system.

**Figure 1:**
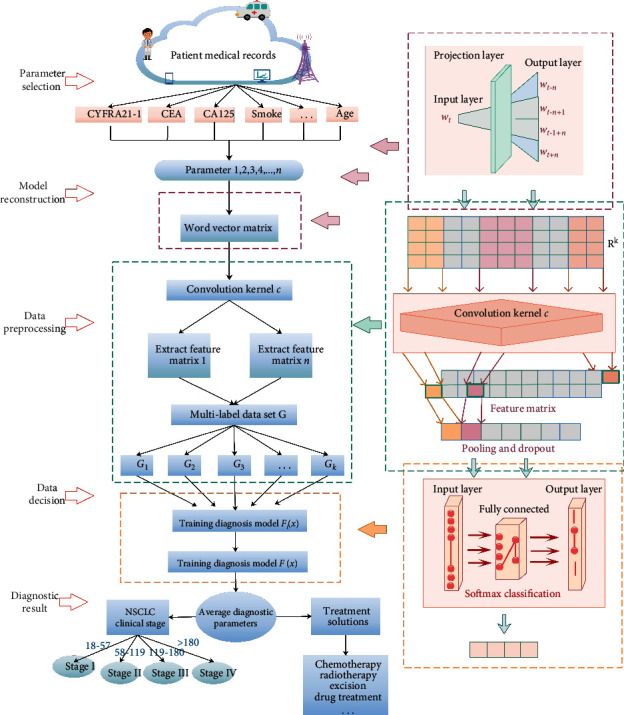
The overall structure of the nonsmall cell lung cancer staging diagnosis system.

We model the process of the auxiliary diagnosis model through five stages (Figure 1): parameter selection, machine learning model (MLM) reconstruction, data preprocessing, data decision-making, and finally, return the auxiliary diagnosis result. Each module in the intelligent medical system has independence and concurrency, so the system can diagnose multiple cases at the same time and improve the diagnosis efficiency. Each stage is described as follows.

The first stage is to obtain NSCLC patient information. We use the patient's age, genetic factors, smoking or not, and some tumor markers as the system diagnosis parameters.

In the second and third stages, disease indicators are preprocessed and converted into a format understandable by the machine learning model. In the NSCLC patient's medical records, the text is segmented into words and converted into word sequences. The Skip-Gram model is used to convert the discrete word symbols into a word vector matrix expressing NSCLC patients.

In the fourth stage, the system completes the data decision. In the NSCLC staging-assisted diagnosis model, the detection parameters are classified by convolution, pooling, and softmax to obtain a diagnosis decision value. According to the range of this value, the clinical stage of NSCLC patients can be judged.

Finally, in the fifth stage, the diagnosis result is returned. After a series of iterative training, the auxiliary diagnosis system will return a diagnosis decision value. The doctor combined the patient's medical history, auxiliary diagnosis results, and the patient's symptoms to make a second diagnosis, which greatly improved the diagnosis' accuracy.

### 3.2. CNNSAD Model Based on Convolutional Neural Network

The CNNSAD model is based on a convolutional neural network (CNN). CNN [29] can automatically extract semantic features of text for classification. Compared with traditional machine learning models, CNN [30] avoids the cost of manual feature extraction and the effect of model implementation on manual feature extraction. In addition, the CNN model is highly flexible and can adapt to problems of various degrees of difficulty. During the CNNSAD training process, the number of NSCLC stages is limited, the symptoms of each stage are different, and the tasks are complicated. Therefore, it is feasible to use the CNN model to realize the auxiliary diagnosis of NSCLC staging.

#### 3.2.1. Skip-Gram Model

The symptoms, treatment options, and prognosis of NSCLC are different in the early, middle, and late stages, which is very necessary for the diagnosis of lung cancer patients. The test indicators for diagnosis of NSCLC patients include blood routine, tumor markers, blood coagulation function, and liver and kidney function. In the staging diagnosis of lung cancer, the parameters of these indicators are comprehensively considered, and the system can analyze the clinical stage of the patient and assist the doctor in the second diagnosis.

The parameters of lung cancer diagnostic indicators affect each other. We link the discrete parameters, which can effectively prevent the loss of important information. Similar to the conversion of “two cycles of chemotherapy” to “chemotherapy,” the “two cycles” of lost treatment time no longer occurs. The Skip-Gram model is a model that uses word *w*_*t*_ to predict the context word set *CW*(*w*_*t*_) = {*w*_*t*−*n*_, *w*_*t*−*n*+1_, ⋯, *w*_*t*+*n*−1_, *w*_*t*+*n*_} in window *n*. *n* will affect the training time and accuracy of the results. The model is trained by maximizing the log-likelihood function to obtain a vector representation of each parameter.

CNNSAD uses the Skip-Gram model to pretrain word vectors of words on patient-related text data and represents discrete word symbols as semantic vectors in low-dimensional continuous space. During training, we need to loop each word as the central word to infer the context. Finally, we can infer the adjacent 2*n* parameters based on a central NSCLC auxiliary diagnostic parameter.

The maximized log likelihood function is

(1) $$\begin{array}{c} L=\underset{\delta \in CW\left(w_{t}\right)}{\sum }\log p\left(\delta \;|\;w_{t}\right). \end{array}$$

*w*_*t*_ is the center, and *CW*(*w*_*t*_) is the corpus. *δ* represents the set of context words to be diagnosed in *CW*(*w*_*t*_).

#### 3.2.2. CNNSAD Model Based on a Convolutional Neural Network.

The CNNSAD model proposed in this paper includes a convolutional layer and a pooling layer. First, performing a convolution operation on the two-dimensional feature matrix, where the length of the convolution kernel is consistent with the length of the word vector, and each convolution kernel generates a column vector representation. The model then uses the maximum pooling method to select the maximum value in each column vector as the output and to form a fixed-dimensional vector of the maximum value of all column vectors. The vector's length is consistent with the number of convolution kernels and is called a feature vector. Finally, the feature vector is input to the fully connected classification layer for patient classification.

The detection of tumor markers in patients with lung cancer is of great significance for early diagnosis, observation of curative effects, and prognosis monitoring. The main tumor markers related to nonsmall cell lung cancer include the Cytokeratin (CYFRA21-1), Carcinoembryonic antigen (CEA), and Cancer antigen125 (CA125). For NSCLC patients, the values of these parameters will be higher. Therefore, we set CYFRA21-1, CEA, and CA125 as the main parameters. By monitoring these parameters' values in the patient's medical record, it is possible to determine whether the patient has lung cancer preliminarily.

In addition, the patient's age, smoking habits, etc. will also affect the effectiveness of decision-making. In order to improve the accuracy of diagnosis and the effect of detection and treatment, we must consider more detection indicators. We set an average diagnostic parameter, the staging auxiliary diagnostic value *F*(*x*) to evaluate the severity of NSCLC. This value takes into account multiple tumor markers and other detection indicators.

Assuming that the model uses a *k*-dimensional vector to represent the word vector, and *x*_*i*_ ∈ *R*^*k*^ represents the word vector representation of the *i*-th parameter. The patient's medical record case contains *n* parameters, and the NSCLC patient's medical record can be represented as *X*_1:*n*_, where ⊕ represents the vector connection operation, as shown in the following equation.

(2) $$\begin{array}{c} X_{1:n}=x_{1}⊕x_{2}⊕\cdots ⊕x_{n}. \end{array}$$

After the two-dimensional matrix representation of the text is generated, the result will be input to the convolutional layer, and the convolution kernel is used to extract some semantic features in the training data. Given (1) *X*_*i*:*i*+*m*−1_ represents the window vector-matrix from the *i*-th parameter to the (*i* + *m* − 1)-th parameter in the word sequence; (2) a convolution kernel matrix *c* ∈ *R*^*k*^, whose purpose is to apply *c* to *m* continuous word vectors to produce an output result.

**Figure 2:**
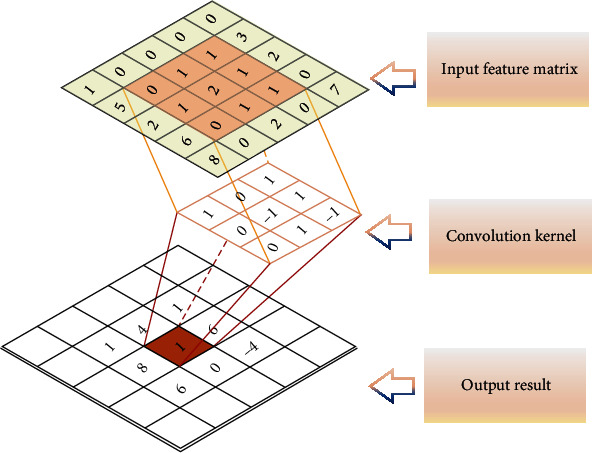
Convolution operation diagram.

Figure 2 shows a schematic diagram of the convolution operation. The result *δ*_*i*_ produced by the convolution kernel *c* acting on *X*_*i*:*i*+*m*−1_ can be calculated:

(3) $$\begin{array}{c} \delta_{i}=f\left(c\cdot X_{i:i+m-1}+a\right), \end{array}$$

where *f*(∗) is a function, *c* is the above convolution kernel, and *a* is a bias term.

For the diagnosis of NSCLC patients, CYREA21-1, CEA, and CA125 are the most important tumor markers in the diagnosis of NSCLC. According to these three detection parameters, the patient's health status can be preliminarily judged and NSCLC patients can be classified. However, other testing indicators will also affect the decision-making results, such as the age of the patient and whether or not to smoke. Studies have shown that if a person smokes heavily for a long time, the probability of developing lung cancer will increase by more than 10 times.

In order to make the decision more accurate, multiple convolution kernels with different windows are used in the model to obtain more parameter information. After passing through the convolutional layer, a feature map whose dimension changes with the sentence length is generated. Patients with NSCLC have complex conditions. The feature matrix obtained from the parameters has a large dimension, and it is difficult to directly train a suitable classification model. Therefore, these feature maps are used as the pooling layer's input to reduce the dimension and capture the most important NSCLC information. As shown in Figure 1, the model in this paper uses the maximum pooling method. Maximum pooling outputs the maximum value in the feature map as the result. After passing through the pooling layer, a fixed-length feature vector (the length is the same as the number of convolution kernels) is generated, and the feature vector is input to the fully connected classification layer for sample classification.

### 3.3. CNNSAD Model Combining Dynamic Sampling and Transfer Learning

This article uses 8920 NSCLC patients as samples. When assisting diagnosis, the positive sample set is the patient data of the NSCLC stage, and the negative sample set is the patients of all other stages. The number of negative samples is much larger than the number of positive samples. Moreover, it is difficult to detect NSCLC at an early stage. Many patients are already in the local middle and advanced stages when they are diagnosed. There are more patients in NSCLC III and IV stages. Therefore, various types of training data are not balanced. The imbalance of training data directly affects the recall rate of the model. In order to improve the impact of the imbalance in the sample size of patients with different stages of NSCLC on the performance of the model, the CNNSAD model proposed dynamic sampling and fusion into the training of the model. At the same time, it is proposed to use transfer learning technology to improve the performance and convergence speed of NSCLC diagnosis.

In order to train the model effectively on the small sample NSCLC data, we first choose the large sample staging that has a high frequency of cooccurrence with this NSCLC staging and train the efficient staging auxiliary diagnosis model on the large sample staging with sufficient data set. Then, we use the large-sample staging assistant diagnosis model as the initial value of the small sample model and retrain the NSCLC staging assistant diagnosis model on the small sample data set. Simultaneously, when NSCLC staging aids diagnosis, the size and extent of tumor spread are very complicated. Therefore, after each iteration of training, we need to update the sample sampling frequency based on the model's diagnostic results on the sample set, increase the sampling frequency of incorrectly classified and low-confidence samples, and then construct a balanced training data set training model through dynamic sampling. In order to ensure the balance between the number of positive samples and the number of negative samples in the selected data set, the model samples the positive samples and negative samples separately. Finally, the sampled NSCLC patient data sets are merged as the next round of training data. In order to improve the effect of transfer learning, the frequency of cooccurrence between two tags is calculated, and the NSCLC staging model with the most cooccurrence is selected as the initialization model. This paper proposes a staging-assisted diagnosis model of NSCLC that combines transfer learning and dynamic sampling. The whole process is shown in Algorithm 1.

(1) Source model training

(a) For any label *δ*_*i*_, calculate the cooccurrence frequency of NSCLC staging labels *δ*_*i*_ and *δ*_*j*_ is calculated

(4) $$\begin{array}{c} T\left(\delta_{i},\delta_{j}\right)=\underset{\left(x_{n},y_{n}\right)\in G}{\sum }E\left(\left\{\delta_{i},\delta_{j}\right\}\subseteq y_{n}\right), \end{array}$$

(5) $$\begin{array}{c} E\left(\left\{\delta_{i},\delta_{j}\right\}\subseteq y_{n}\right)=\left\{\begin{array}{cc} 1, & \delta_{i}\in y_{n}\text{ and }\delta_{j}\in y_{n},\\ 0, & \delta_{i}\notin y_{n}\text{ or }\delta_{j}\notin y_{n}. \end{array}\right. \end{array}$$

Among them, *T* is the cooccurrence frequency of NSCLC staging labels *δ*_*i*_ and *δ*_*j*_. If any label *δ*_*i*_ and *δ*_*j*_ cooccur at a certain moment, then *E* = 1. On the contrary, if there is no cooccurrence at this moment, then *E* = 0.

(b) Using the One-Vs-Rest method to split G into multiple stages of the two-class NSCLC patient data set {G_1_, G_2_, ⋯, G_*K*_}. Where G_*i*_ is the training set of the NSCLC staging label *δ*_*i*_. Selecting the staging label *δ*_*i*_ that cooccurs most frequently with the NSCLC staging label *δ*_*k*_ and train the staging auxiliary diagnosis model *F*_*k*_(*x*) on the training data set *G*_*k*_ of *δ*_*k*_

(6) $$\begin{array}{c} F_{i}\left(x\right)=\text{train}\left(G_{k}\right). \end{array}$$

Parameter *F*_*i*_(*x*) is the average diagnostic parameter of NSCLC. After training, the decision result is the value of the average diagnostic parameter. We will save the *F*_*i*_(*x*) parameter. By calculating the value of the parameter *F*_*i*_(*x*), it is possible to determine which stage of the NSCLC patient's condition is, so as to choose an effective treatment method.

When the value of *F*_*i*_(*x*) is greater than 18 and less than 57, the patient is in NSCLC stage I; when the value of *F*_*i*_(*x*) is greater than 57 and less than 119, the patient is in NSCLC stage II. In the first two stages, doctors can use medication or surgery or a combination of both. When the value of *F*_*i*_(*x*) is greater than 119 and less than 180, the patient is in NSCLC III; when the value of *F*_*i*_(*x*) is greater than 180, the patient is in NSCLC IV.

(2) Small sample NSCLC staging training stage. When the patient is in the latter two stages, doctors can use radiotherapy or chemotherapy or a combination of both

(a) Read the parameters of the deep learning model *F*_*k*_(*x*) as the initialization model *F*_*i*,1_(*x*) of the NSCLC staging label *δ*_*i*_. The most category data set of *δ*_*i*_ is *G*_*k*,neg_, the minority category data set is *G*_*k*,pos_, the number of patients is *N*_neg_ and *N*_pos_, and the total data volume is *N*. Initialize the sampling probability *ρ*_*i*,1_ = {*ρ*_*i*,1_(1), *ρ*_*i*,1_(2), ⋯, *ρ*_*i*,1_(*N*), }of minority patients

(7) $$\begin{array}{c} \rho_{i,1}\left(j\right)=\left\{\begin{array}{c} \frac{\text{size}}{2\times N_{\text{pos}}},\;\delta_{i}\in y_{j},\\ \begin{array}{cc} \frac{\text{size}}{2\times N_{\text{neg}}}, & \delta_{i}\notin y_{j}. \end{array} \end{array}\right. \end{array}$$

The sum of the probabilities of the positive and negative samples is size/2. After sampling each positive and negative sample according to the following sampling method (b), the average number of positive and negative samples obtained by sampling is size/2. Therefore, the sample constructed by sampling is balanced.

(b) Sample sampling based on patient sampling probability *ρ*_*i*,*t*_, and sample the positive sample set and the negative sample set, respectively. For any sample (*x*_*j*_, *y*_*j*_), its sampling probability is *ρ*_*i*,*t*_(*j*). Using Random(*x*) to randomly generate a uniformly distributed value Random(*x*_*j*_) between 0-1. When Random(*x*_*j*_) < *ρ*_*i*,*t*_(*j*), the sample (*x*_*j*_, *y*_*j*_) is added to the new balanced sample set *G*_*i*,train_. At this time, if *δ*_*i*_ ∉ *y*_*j*_, add sample (*x*_*j*_, *y*_*j*_) to the sample set *G*_*i*,neg_^set^ of majority cases; if *δ*_*i*_ ∈ *y*_*j*_, add sample (*x*_*j*_, *y*_*j*_) to part of the sample set *G*_*i*,pos_^set^ of minority cases

(8) $$\begin{array}{c} G_{i,\text{neg}}^{\text{set}}=\left\{\left(x_{i},y_{i}\right)\;|\;\text{Random}\left(x_{j}\right)\leq \rho_{i,t}\left(j\right),\left(x_{j},y_{j}\right)\in G_{i,\text{neg}}\right\}, \end{array}$$

(9) $$\begin{array}{c} G_{i,\text{pos}}^{\text{set}}=\left\{\left(x_{i},y_{i}\right)\;|\;\text{Random}\left(x_{j}\right)\leq \rho_{i,t}\left(j\right),\left(x_{j},y_{j}\right)\in G_{i,\text{pos}}\right\}. \end{array}$$

For each sample (*x*_*j*_, *y*_*j*_), its sampling frequency is *ρ*_*i*,*t*_(*j*), which is equal to the probability that the randomly generated number Random(*x*_*j*_) is less than *ρ*_*i*,*t*_(*j*). When Random(*x*_*j*_) is less than *ρ*_*i*,*t*_(*j*), sample (*x*_*j*_, *y*_*j*_) is added to the balanced case sample set. So it is reasonable to use this algorithm to update the sampling frequency. Finally, *G*_*i*,neg_ and *G*_*i*,pos_ form the training set *G*_*i*,train_:

(10) $$\begin{array}{c} G_{i,\text{train}}=G_{i,\text{neg}}^{\text{set}}\cup G_{i,\text{pos}}^{\text{set}}. \end{array}$$

(c) *F*_*i*,*t*−1_(*x*) is trained based on data set *G*_*i*,train_ to generate a new model *F*_*i*,*t*_(*x*)

(11) $$\begin{array}{c} F_{i,t}\left(x\right)=\text{train}\left(F_{i,t-1}\left(x\right);G_{i,\text{train}}\right). \end{array}$$

(d) Calculating the probability that the diagnostic sample of model *F*_*i*,*t*_(*x*) on the overall training sample is a positive sample is *P*_*i*,*t*_. *P*_*i*,*t*_(*j*) ∈ [0, 1], represents the probability value that the diagnostic sample belongs to the positive sample. For positive samples, the larger *P*_*i*,*t*_(*j*) is, the better, for negative samples, the smaller *P*_*i*,*t*_(*j*) is, the better. Using *P*_*i*,*t*_ to update the sampling probability *ρ*_*i*,*t*+1_ = {*ρ*_*i*,*t*+1_(1), *ρ*_*i*,*t*+1_(2), ⋯, *ρ*_*i*,*t*+1_(*N*), }

(12) $$\begin{array}{c} P_{i,t+1}\left(j\right)=\left\{\begin{array}{c} \begin{array}{cc} \rho_{i,t}\left(j\right)\exp\left(1-P_{i,t}\left(j\right)\right), & \delta_{i}\in y_{j}, \end{array}\\ \begin{array}{cc} \rho_{i,t}\left(j\right)\exp\left(P_{i,t}\left(j\right)\right), & \delta_{i}\notin y_{j}. \end{array} \end{array}\right. \end{array}$$

The differences in different stages of NSCLC lead to the imbalance and small sample characteristics of patient samples. Therefore, we need to adopt appropriate strategies to generate a balanced data set. When training case samples, the model uses the diagnosis results on different samples to dynamically update the sampling probability, ensuring that more attention is paid to misclassified patients and patients with low classification confidence to improve the diagnosis.

The method to update the sampling probability is when the model *F*_*i*,*t*_(*x*) diagnoses NSCLC patients with a wrong sample or the diagnosis is correct and the diagnosis confidence is not high, increase the sampling probability of the sample, thereby increasing the model's attention to the sample; conversely, relatively reduce the sample's probability of sampling and reduce the model's attention to the sample. This can increase the distinguishability of the model for positive and negative samples and improve the diagnostic accuracy and confidence of the model. Therefore, when sample (*x*_*j*_, *y*_*j*_) is a positive sample, the closer *P*_*i*,*t*_(*j*) is to 0, the classification is wrong, or the classification is correct, but the confidence is not high, and the updated sampling probability increases. Conversely, when it is a negative sample, the closer *P*_*i*,*t*_(*j*) is to 1, the classification is wrong, or the classification is correct, but the confidence is not high, and the updated sampling probability increases.

It is regularizing the sampling probability of positive *H*_*t*,pos_ is the sum of the sampling probabilities of positive samples.

(13) $$\begin{array}{c} \rho_{i,t+1}\left(j\right)=\frac{\text{size}\times \rho_{i,t+1}\left(j\right)}{2\times H_{t,\text{pos}}}, \end{array}$$

(14) $$\begin{array}{c} H_{t,\text{pos}}=\underset{\left(x_{n},y_{n}\right)\in G_{i,\text{pos}}}{\sum }\rho_{i,t+1}\left(n\right). \end{array}$$

The sampling probability of negative samples is regularized, where *H*_*t*,neg_ is the sum of the sampling probabilities of all negative samples.

(15) $$\begin{array}{c} \rho_{i,t+1}\left(j\right)=\frac{\text{size}\times \rho_{i,t+1}\left(j\right)}{2\times H_{t,\text{neg}}}, \end{array}$$

(16) $$\begin{array}{c} H_{t,\text{neg}}=\underset{\left(x_{n},y_{n}\right)\in G_{i,\text{neg}}}{\sum }\rho_{i,t+1}\left(n\right). \end{array}$$

(e) Determining whether the specified number of iterations is reached, and if it is satisfied, return to the final classifier; otherwise, use the new sampling probability to proceed to steps (b) ~ (e)

After multiple iterations of training, the sampling frequency is constantly updated to make the decision value more accurate. The auxiliary system will judge the stage of NSCLC patients according to the range of decision values. So as to assist the doctor in analyzing the patient's condition.

## 4. Experiments and Conclusions

This article collects and collates data on NSCLC patients with different clinical stages in three hospitals in China from 2011 to 2015, as shown in Table 1. We use CYFRA21-1, CEACA125, squamous cell carcinoma antigen (SCC), carbohydrate antigen 15-3 (CA15-3), and carbohydrate antigen 19-9 (CA19-9) six tumor markers for diagnosis experiment analysis. According to clinical medical standards, Table 2 shows the six diagnostic parameters of NSCLC and their normal ranges. We input the samples of diagnosed nonsmall cell lung cancer patients into the system and calculate the average diagnostic parameter value range of each stage of NSCLC, as shown in Table 3.

**Figure 3:**
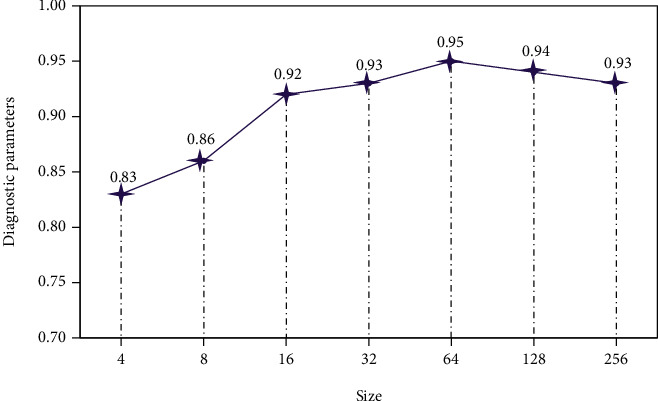
Data block size.

The choice of training samples will affect the sampling frequency and cause the bias of prediction.  Figure 3 shows the relationship between the size of the data block and the accuracy of the evaluation index at each iteration. As the size increases, the accuracy of the diagnostic parameters first increases and then decreases. When size = 64, that is, when there are 64 samples in each block, the accuracy reaches the highest value. Therefore, in the iterative training of the model in this paper, we choose size = 64 to train the staging auxiliary diagnosis system.

### 4.1. Algorithm Performance Analysis

In order to evaluate the performance of the CNNSAD classification algorithm, we selected four classification algorithms: lasso regression (LASSO) [29], decision tree (DT), support vector machine (SVM), and *k*-nearest neighbor (k-NN) for comparison analysis. The experiment uses the average value of 10-fold cross-validation as the prediction result to ensure the accuracy of the experimental results. Among them, precision (Pre), recall (Re), accuracy (Acc), and AUC value (Area Under ROC Curve) are used as criteria to evaluate the performance of different classification algorithms.

According to the true cases (TP), false positive cases (FP), true negative cases (TN), and false negative cases (FN) in the confusion matrix [30], the values of Pre, Re, Acc, and AUC can be calculated.

(17) $$\begin{array}{c} \text{Pre}=\frac{\text{TP}}{\text{TP}+\text{FP}}, \end{array}$$

(18) Re=TPTP+FN,

(19) $$\begin{array}{c} \text{Acc}=\frac{\text{TP}+\text{FN}}{\text{TP}+\text{TN}+\text{FP}+\text{FN}}. \end{array}$$

**Figure 4:**
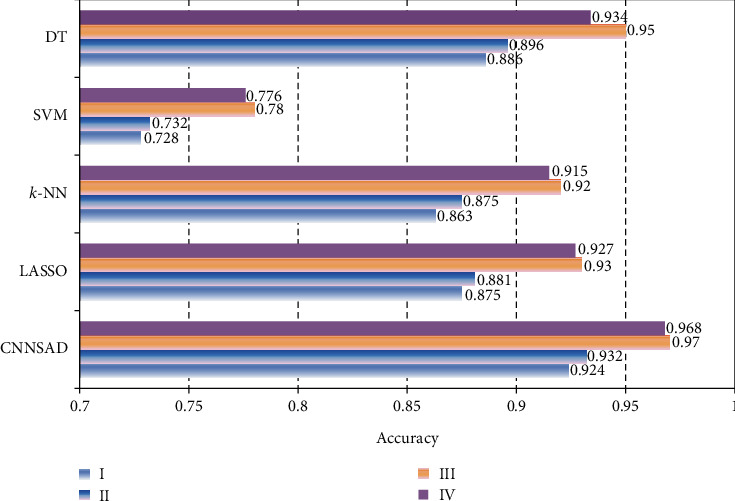
The accuracy of the algorithm.

Figure 4 shows the accuracy levels of several classification algorithms in NSCLC staging. It can be seen from the figure that the accuracy of the SVM classification algorithm is the lowest. LASSO and *k*-NN are more accurate when the patient is in stage III or IV, but when the patient is in NSCLC stage I or II, the accuracy is significantly reduced. *k*-NN only calculates the “nearest” neighbor samples, so the accuracy of *k*-NN increases relative to SVM. Lasso solves the multiple linearity problems in regression and improves the accuracy of classification. The accuracy of CNNSAD reaches 0.97 when diagnosing whether the patient is in NSCLC stage III, and it is always higher than other algorithms in other stages, indicating that this model has higher accuracy in diagnosing the clinical stage of NSCLC patients.

Table 4 shows the number of examples of incorrect classification of the CNNSAD algorithm. It can be seen that the patient is more likely to be misdiagnosed as an adjacent clinical stage.

**Figure 5:**
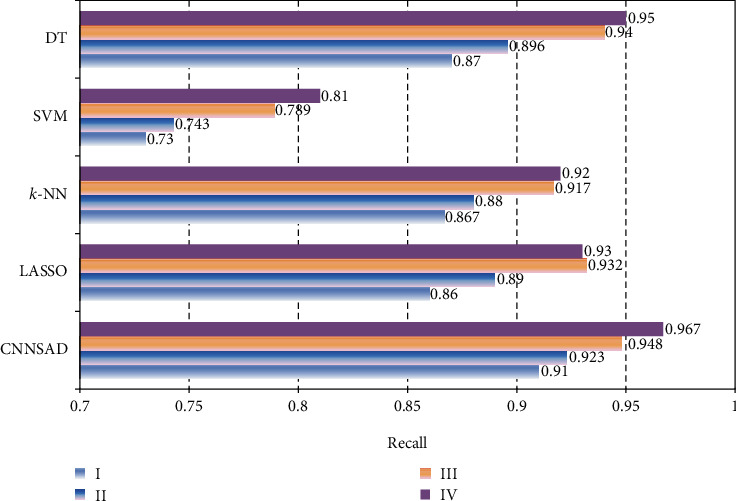
Algorithm recall rate.

**Figure 6:**
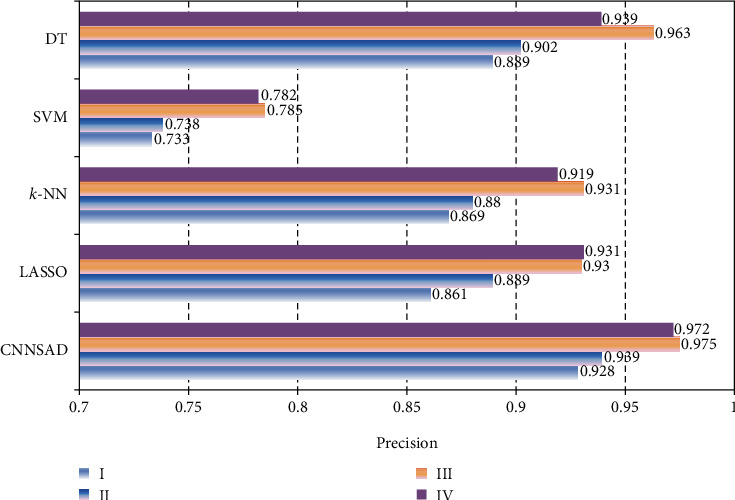
Algorithm precision rate.

Figure 5 shows the recall levels of several classification algorithms.  Figure 6 shows the precision of several classification algorithms. As can be seen from the figure, CNNSAD always occupies the highest point. It shows that CNNSAD has the best performance regardless of the diagnosis result or the sample. Through comparative analysis, we can know that the CNNSAD model has outstanding advantages in finding the right (predicting the clinical stage of the patient) and finding the full (finding the clinical stage of all patients) when performing NSCLC staging auxiliary diagnosis. This is because CNNSAD uses convolutional networks to automatically extract text features, which reduces the impact of noise and greatly improves its classification performance. At the same time, the combination of dynamic sampling and transfer learning technology improves the impact of unbalanced training data. Therefore, the recall and precision of the model are improved.

**Figure 7:**
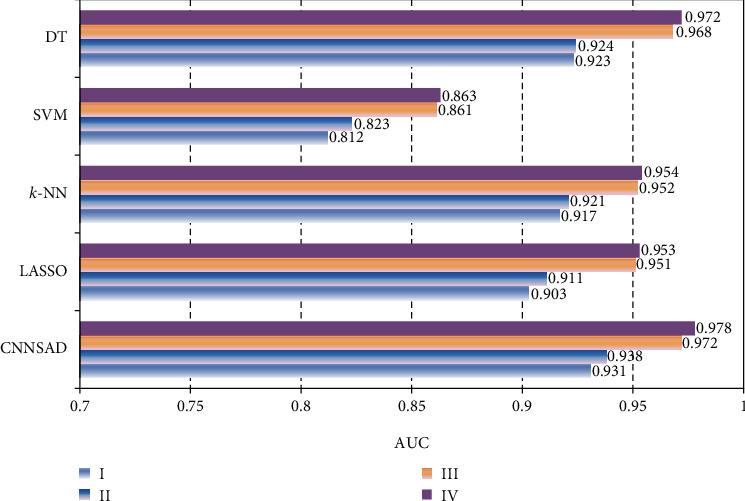
The AUC value of the algorithm.

Figure 7 shows the AUC values of several classification algorithms in different stages of NSCLC. The SVM model, which is very difficult to train, has low indicators for all four stages, always below 0.9. Compared with SVM, *k*-NN has better performance. It can be seen from the figure that although the DT classifier has a higher AUC value in NSCLC III or IV, DT has a large fluctuation in several NSCLC staging classifications. Therefore, this model is not suitable for the staged diagnosis of NSCLC patients. The lasso classification algorithm reduces the variability in regression and improves the accuracy of the model, so the AUC value is higher. It can be seen from the figure that the value of CNNSAD is always the largest, indicating that this method has the best performance, and the effect of feature classification is better.

In summary, compared to several other classification algorithms in the experiment, the CNNSAD method proposed in this paper has better performance. Especially when NSCLC patients are in stage I or II, the classification accuracy is higher.

### 4.2. NSCLC Data Analysis and Decision Making

**Figure 8:**
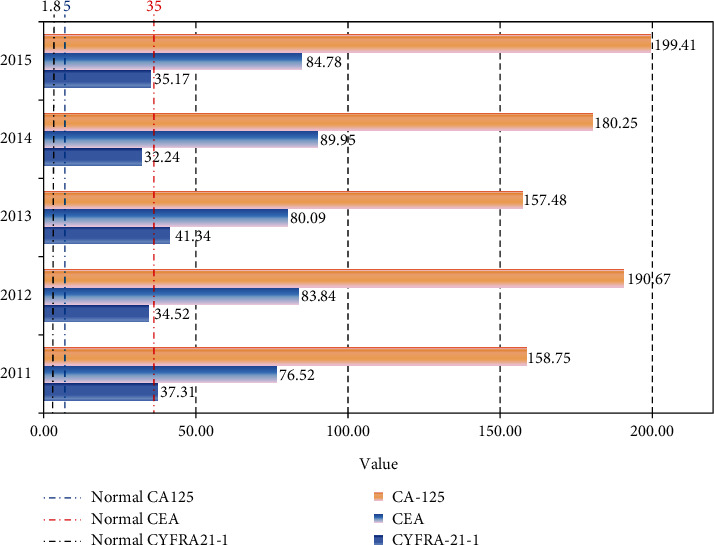
Average performance of diagnostic parameters.

Figure 8 shows the average performance of the diagnostic parameters of NSCLC patients in the three hospitals from 2011 to 2015. As can be seen from the figure, the range of CYFRA21-1 for healthy people is 0 to 1.8, the normal range for CEA is 0 to 5, and the normal range for CA125 is 0 to 35. The sampling results of CYFRA21-1 of NSCLC patients in the past five years have exceeded 35 on average. The average CEA sampling result is around 80, which is 16 times the normal value. The average CA125 value reaches 175. All three are far beyond their normal values. It shows that CYFRA21-1, CEA, and CA-125 of NSCLC patients are in an abnormal state.

**Figure 9:**
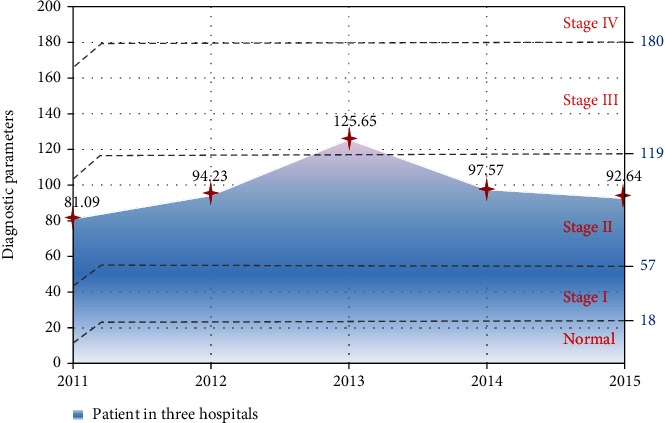
Average decision parameters of NSCLC staging in three hospitals.

The treatment options and prognosis of lung cancer are different in the early, middle, and late stages. Determining the clinical stage of the patient is the key to choosing the best treatment.  Figure 9 shows the decision-making parameters of NSCLC staging in three hospitals in the last five years. From 2011 to 2013, the average decision-making parameter values of patients in the three hospitals continued to rise. It even increased to 125.65 in 2013, 1.5 times that of 2011. After 2013, the average value of decision-making parameters continued to decline, falling to 92.64 in 2015. The average value of decision parameters in the five years is around 95, indicating that most patients with NSCLC are in the second stage.

**Figure 10:**
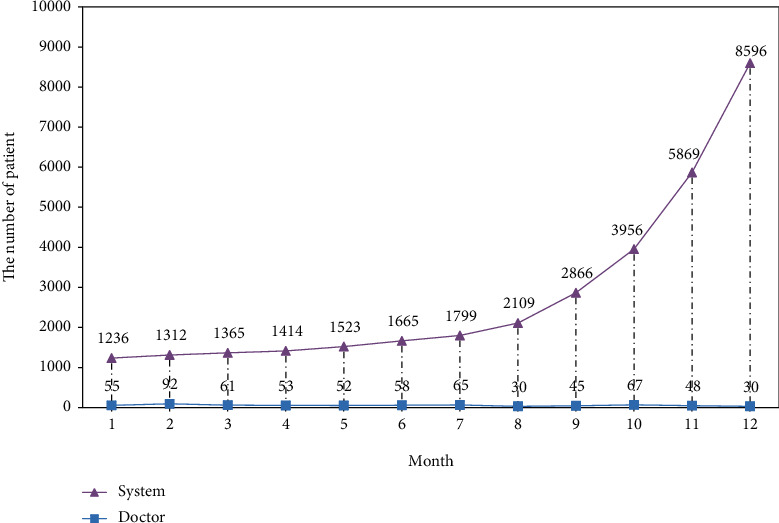
The number of patients diagnosed by doctors and the system each month.

**Figure 11:**
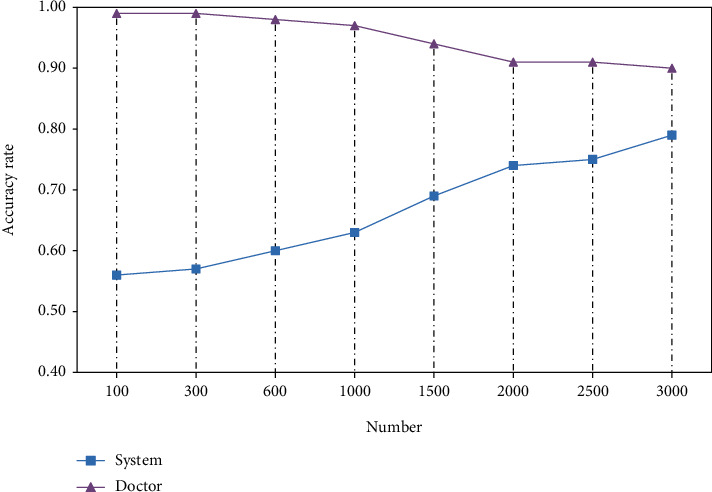
The efficiency of doctors and systems in diagnosing patients.

The number and efficiency of doctors and the system diagnosed patients in a year are shown in Figures 10 and 11, respectively. It can be seen from Figure 10 that the number of patients diagnosed by doctors per month does not change much, remaining at about 50. The number of diagnoses made by the diagnostic system every month continues to rise. In the first 7 months, due to the lack of training data, a new data set needs to be entered manually, and the number of diagnoses per month of the system is less than 2000. As the medical data learned by the system continues to increase, the efficiency of the system's medical treatment has been greatly improved. By the end of the year, the number of diagnoses per month can reach 8,500.

The diagnostic accuracy of the auxiliary diagnostic system is also very important. As shown in Figure 11, we compared the diagnostic accuracy of the doctor and the assistant system. When the patient data is less than 500, the doctor's diagnosis accuracy is very high, always reaching 99%. With the increase in patient data, the accuracy has declined, but it is always higher than 90%. When the patient data is small, the accuracy of the machine-assisted system is less than 70%. As patient data increases, the accuracy rate increases. When there are more patient data, it can reach 90%.

Although there is a big gap between the results of the decision-making system and the doctor's diagnosis, the system's diagnosis speed is very fast. In actual medical decision-making, we can use it to assist the doctor. Especially when there is a lot of patient data, it can effectively reduce the workload of the doctor and improve the diagnosis efficiency.

## 5. Conclusion

This paper proposes a staging-assisted diagnosis model for NSCLC and uses the data and information of 8,920 NSCLC patients collected from three hospitals in China for simulation experiments. The CNNSAD model converts the patient's medical record into a sequence of words and uses the convolutional neural network to extract semantic features from the patient's medical record to train the model to assist in the diagnosis of NSCLC. At the same time, CNNSAD combines migration learning and dynamic sampling technology to effectively solve the impact of imbalanced case samples on model training and diagnostic performance, thereby improving the diagnostic performance of the model. Doctors use the diagnosis result of the auxiliary system as a reference for the second diagnosis, which can greatly improve the accuracy of diagnosis and work efficiency.

With the development of smart medical care, in the future, we will deeply optimize the diagnosis model and training algorithm to achieve better staging assisted diagnosis. At the same time, we will further study the heuristic diagnosis method based on the knowledge map in the medical field and use a variety of information in the knowledge map to improve the interpretability and diagnostic accuracy of the model and provide doctors with scientific and effective data analysis and treatment plans.

## Figures and Tables

**Algorithm 1:**
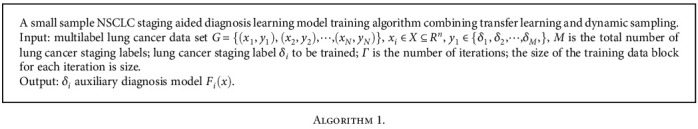


**Table 1:** NSCLC patient data from three hospitals.

Stage	Stage I	Stage II	Stage III	Stage IV
Number	752	1497	3926	2745

**Table 2:** Diagnose parameter and decision data with normal data in NSCLC.

Landmark	Range (ng/ml)
CYFRA21-1	0-1.80
CEA	0-5.00
CA125	0-35.00
SCC	0-1.50
CA19-9	0-37
CA15-3	0-30

**Table 3:** Average diagnostic parameter range of each stage of NSCLC (age 35–78 years).

Stage I	18-57
Stage II	58-119
Stage III	119-180
Stage IV	>180

**Table 4:** CNNSAD algorithm diagnosis.

	Classification errors	Sample number distribution			
		I	II	III	IV
Stage I	57		36	14	7
Stage II	102	43		49	10
Stage III	118	1	55		62
Stage IV	288	2	33	53	

## Data Availability

The data used to support the findings of this study are currently under embargo while the research findings are commercialized. Requests for data, 12 months after publication of this article, will be considered by the corresponding author.
